# Cancer Therapy-Related Cardiotoxicity: A Comprehensive Retrospective Analysis at Najran Cancer Center, Saudi Arabia

**DOI:** 10.7759/cureus.41287

**Published:** 2023-07-02

**Authors:** Ahmed M Badheeb, Faisal Ahmed, Hassan A Alzahrani, Mohamed A Badheeb, Hamoud Y Obied, Islam A Seada

**Affiliations:** 1 Oncology, Oncology Center, King Khalid Hospital, Najran, SAU; 2 Urology, Ibb University, Ibb, YEM; 3 Internal Medicine, King Khalid Hospital, Najran, SAU; 4 Internal Medicine, Yale New Haven Health, Bridgeport Hospital, Bridgeport, USA; 5 Cardiac Surgery, King Khalid Hospital, Najran, SAU; 6 Extracorporeal Membrane Oxygenation (ECMO), ECMO Program, King Khalid Hospital, Najran, SAU; 7 Cardiothoracic Surgery, Mansoura University, Mansoura, EGY

**Keywords:** saudi arabia, najran, cardiac toxicity, chemotherapy, cancer

## Abstract

Background

Cardiotoxicity, produced as an adverse effect of anticancer therapy, is a common issue during cancer treatment. Acute coronary syndrome, myocarditis, arrhythmias, or heart failure can all be symptoms of this issue. Little is known about its occurrence among Saudi Arabian cancer patients. This study aims to investigate factors linked to anticancer therapy-related cardiotoxicity.

Methods

A retrospective study was conducted from April 2020 to May 2022 at the King Khalid Hospital, Najran, Saudi Arabia. The study included adult cancer patients receiving anticancer therapy, regardless of their cardiovascular disease history. Univariate analysis was used to investigate factors associated with the occurrence of cardiotoxicity related to anticancer therapy.

Results

Of 78 patients receiving anticancer therapy, cardiotoxicity occurred in 12 (15.4%) patients. The mean age was 56.5 ± 13.4 years, with 33.3% aged over 65 years. Comorbidities included hypertension (44; 56.4%), diabetes (41; 52.6%), dyslipidemia (13; 16.7%), smoking (16; 20.5%), heart disease (6; 7.7%), trastuzumab use (9; 11.5%), and chronic kidney disease (2; 2.6%). The most common cancers were breast cancer and gastrointestinal cancer (27.6% each). Monoclonal anticancer agents 35 (46.1%) and alkylating agents 29 (38.2%) were commonly used chemotherapies. Cardiac protective agents were used in 16 (21.1%) of patients, with angiotensin-converting enzyme (ACE) inhibitors 15 (19.7%) and statins (13; 17.1%) being the most prescribed. Baseline ejection fraction (EF) was normal in 69 (90.8%) of cases. The follow-up duration was 1.93 ± 1.90 years. A drop in EF occurred in five (6.6%) of cases. Dyslipidemia (OR: 0.12; 95% CI: 0.03-0.47, p=0.002), previous heart disease (OR: 0.14; 95% CI: 0.02-0.81, p=0.029), and impaired baseline EF (p=0.029) were associated with increased risk of cardiotoxicity. Statin (OR: 0.22; 95% CI: 0.05 to 0.84, p=0.028) and antiplatelet agents (OR: 0.19; 95% CI: 0.03 to 1.01, p=0.051) were protective agents against cardiac toxicity.

Conclusion

Effective anti-cancer therapy may be accompanied by an increased risk of cardiotoxicity. In this study, a history of prior heart disease, dyslipidemia, low baseline ejection fraction, and the administration of multiple anticancer therapy agents was associated with cardiotoxicity. Proactive management strategies aimed at mitigating the potential cardiotoxic effects of anti-cancer therapies are crucial.

## Introduction

The cardiotoxic effects of anticancer therapy were initially evaluated in late 1970, as an increased incidence of heart failure was observed among cancer patients following the initiation of anthracycline-based chemotherapy [1]. Such an issue became even more apparent with the advancement of cancer screening and detection, and the earlier, and sometimes longer, use of chemotherapeutics among cancer patients, which led inevitably to increased anticancer therapy-related adverse events [2].

Cancer therapies include various drug agents such as molecular target therapies, cytotoxic chemotherapy, and mediastinal irradiation [3]. There are various pathophysiological mechanisms, depending on the drug group, by which anticancer may affect the normal structure or function of the heart such as myocyte damage, ischemia, conduction, rhythm disturbances, left ventricular dysfunction, cardiac failure, and several other cardiovascular complications [4-6]. The most prevalent chemotherapeutic treatments connected to significant cardiac events include anthracyclines, alkylating agents (cyclophosphamide, cisplatin), and taxanes (paclitaxel, docetaxel) [3].

The risk factors of cardiovascular disease (CVD) and cancer suggest a possible overlapped pathogenesis [7]. Aging, physical inactivity, smoking, hypertension, diabetes mellitus, and inflammation appear to contribute to the progression and advancement of both entities [8].

The European Society of Cardiology provided a set of thorough recommendations regarding the pre-treatment assessment, comorbidities management, and long-term surveillance of cancer patients receiving cardiotoxic drugs [9]. Nevertheless, the complexity of patients necessities interdisciplinary cardio-oncology cooperation to provide the possible outcomes [10].

The precise incidence of cardiac toxicity and the extent of adherence to cardiac monitoring recommendations among cancer patients in Saudi Arabia are areas of limited knowledge [8]. Consequently, this study aims to investigate the factors associated with the occurrence of cardiotoxicity among adult cancer patients who receive anticancer therapy at the oncology center of King Khalid Hospital in Najran, Saudi Arabia. By elucidating these factors, we aim to contribute to the existing academic understanding of cardiotoxicity in the context of anticancer treatments and provide valuable insights for clinical practice and patient care.

## Materials and methods

Study design

A retrospective study was conducted at the oncology center of King Khalid Hospital in Najran, Saudi Arabia, focused on anticancer-induced cardiotoxicity in adult cancer patients who underwent anticancer therapy spanning from April 2020 to May 2022. Patients who developed cardiotoxicity continued their remaining therapy, either with the medication removed from the regimen or with a full change of regimen. Ethical approval for the study was obtained from the Ethics Research Committees of King Khalid Hospital, ensuring compliance with the ethical principles stipulated in the Declaration of Helsinki.

Inclusion criteria: This study included adult patients (≥ 18 years) who received anticancer therapy at our center and had undergone left ventricular ejection fraction (LVEF) testing before and after anticancer therapy.

Exclusion criteria: Patients without LVEF test results before or after chemotherapy administration and elderly patients with advanced or terminal diseases were excluded.

The study protocol and main outcome

LVEF assessments were routinely conducted at baseline and three and six months after initiating chemotherapy. Cardiotoxicity was defined based on the criteria established by Guglin et al., which encompassed an LVEF value below 50% or a reduction of LVEF by 10% or more from baseline, along with symptomatic heart failure, even in the absence of a decline in LVEF [9,10]. The primary outcome was the occurrence of cardiac toxicity during the course of chemotherapy. The secondary outcome was to identify the factors associated with cardiotoxicity.

Data collection

Relevant data from electronic records and/or medical charts of eligible cancer patients were collected using a structured data collection format. The collected information included demographic details at the start of anticancer therapy, underlying cardiac conditions, comorbidities, histological cancer type, stage, tumor site, drug regimen, the total number of cycles, administration of cardiac protective agents, concurrent use of cancer chemotherapy, radiotherapy, or endocrine therapy, as well as pre-and post-treatment LVEF values.

Statistical analysis

Descriptive statistics were utilized to present quantitative variables in terms of means and standard deviations while qualitative variables were expressed as frequencies and percentages. The normality of the data was assessed using the Kolmogorov-Smirnov test. To compare patients in the cardiac toxicity and non-cardiac toxicity groups, univariate analysis was performed employing independent samples T-test or Mann-Whitney test for quantitative variables and chi-square or Fisher's exact test for qualitative variables. A significance level of p < 0.05 was considered statistically significant. The statistical analysis was carried out using IBM SPSS version 18 software (IBM Corp., Armonk, New York).

## Results

Among the 78 cancer patients who underwent anticancer therapy, cardiotoxicity was observed in 12 individuals (15.4%). The mean age of the patients was 56.5 ± 13.4 years (ranging from 32 to 81 years), with 26 patients (33.3%) being older than 65 years. A majority of the patients (51; 65.4%) were female. Prevalence rates of hypertension, smoking, dyslipidemia, heart disease, diabetes, trastuzumab use, chronic kidney disease, and previous percutaneous coronary intervention were reported in 44 (56.4%), 16 (20.5%), 13 (16.7%), 6 (7.7%), 41 (52.6%), 9 (11.5%), 2 (2.6%), and 1 (1.3%) patient(s), respectively. The most common types of cancer observed were breast cancer (27.6%) and gastrointestinal cancer (27.6%). A majority of the patients (55.3%) were in the metastatic stage. Monoclonal anticancer agents and alkylating agents were the most frequently used anticancer therapy subgroups, accounting for 35 (46.1%) and 29 (38.2%) cases, respectively. Cardiac protective agents were administered to 16 (21.1%) patients, with ACE inhibitors and statins being the most commonly prescribed for 15 (19.7%) and 13 (17.1%) patients, respectively. The baseline ejection fraction was found to be normal in the majority of cases, with 69 (90.8%) patients exhibiting normal values (Table 1).

**Table 1:** The patient's characteristics Abbreviations: RCVP: rituximab with cyclophosphamide, vincristine, and prednisone; RCHOP: rituximab with cyclophosphamide, doxorubicin, vincristine, and prednisone, AC: doxorubicin and cyclophosphamide; ACE: angiotensin-converting enzyme

Variables	N (%)
Age (year) Mean (SD)	56.5 (13.3) (range 32 - 81)
Age group	
≥65 years	51 (67.1%)
<65 years	25 (32.9%)
Gender	
Male	26 (34.2%)
Female	50 (65.8%)
Smoking	16 (21.1%)
Cardiotoxicity	12 (15.4)
Percutaneous coronary intervention	1 (1.3%)
Hypertension	43 (56.6%)
Diabetes	40 (52.6%)
Heart disease	6 (7.9%)
Chronic kidney disease	2 (2.6%)
Trastuzumab using	9 (11.8%)
Dyslipidemia	13 (17.1%)
Site of cancer	
Lymphoma	8 (10.5%)
Breast	21 (27.6%)
Urologic cancer	8 (10.5%)
Gastrointestinal cancer	21 (27.6%)
Gynecological cancer	7 (9.2%)
Lung	3 (3.9%)
Malignant melanoma	4 (5.3%)
Head and neck cancer	3 (3.9%)
Other cancer	1 (1.3%)
Cancer stage	
Metastatic	42 (55.3%)
Non-metastatic	34 (44.7%)
Use of cardiac protective agents	16 (21.1%)
Cardiac protective agents	
Betablocker	10 (13.2%)
ACE inhibitors	15 (19.7%)
Statins	13 (17.1%)
Antiplatelets	7 (9.2%)
Number of chemotherapy cycles used	
< 5 cycles	33 (43.4%)
Between 6-10 cycles	30 (39.5%)
≥10 cycles	13 (17.1%)
Bassline ejection fraction	
Normal (55 or more)	69 (90.8%)
Between 50 -54% (Borderline low)	1 (1.3%)
Between 36-49% (impaired)	6 (7.9%)
A drop in ejection fraction during treatment	5 (6.6%)
Anticancer therapy protocol	
RCVP	3 (3.9%)
Docetaxel	5 (6.6%)
Folfox	11 (14.5%)
RCHOP	2 (2.6%)
AC	8 (10.5%)
Carbo Taxol	3 (3.9%)
Xelox	5 (6.6%)
Paclitaxel	4 (5.3%)
Other	35 (46.1%)
Anticancer therapy subgroups	
Monoclonal anticancer use	35 (46.1%)
Alkylating agent users	29 (38.2%)
Anthracyclines	16 (21.1%)
Other agents use	2 (2.6%)
Taxane users	11 (14.5%)

Follow-up duration and changes in EF during treatments

The average follow-up duration in this study was 1.93 ± 1.90 years. Out of the patients, five (6.6%) experienced a decrease in ejection fraction (EF) during their treatment period. Interestingly, the decrease in EF was less pronounced among those without cardiac toxicity (Table 2).

**Table 2:** The association between the drop of EF during treatment and factors such as cardiac toxicity occurrence.

Variables	Sub variable	Total (n=78) N (%)	A drop of EF during treatment		Univariate analysis	P-value
			No n (%) 73(93.6)	Yes n (%) 5(6.4)	OR (95 % CI)	
Cardiac toxicity	No	66(84.6)	65(98.5)	1(1.5)	0.03 (0.003 to 0.31)	0.003
	Yes	12(15.4)	8(66.7)	4(33.3)	Reference group	

Risk factors for cardiac toxicity

The univariate analysis revealed that individuals with dyslipidemia had a higher chance of experiencing cardiac toxicity (odds ratio (OR) 0.12; 95% confidence interval (CI) 0.03 to 0.47, p=0.002), as did those with a previous history of heart disease (OR: 0.14; 95% CI: 0.02 to 0.81, p=0.029), and those with impaired baseline EF (p=0.029). Table 3 provides an overview of patient factors and their association with cardiac toxicity (Table 3).

**Table 3:** Patients' factors and their association with cardiac toxicity

Variables	Sub variable	Total (n=78) N (%)	Outcome cardiac toxicity		Univariate analysis	P-value
			No n (%) 66(84.6)	Yes n (%) 12(15.4)	OR (95 % CI)	
Age	<65 years	52(66.7)	46(88.5)	6(11.5)	0.43 (0.12 to 1.51)	0.191
	≥65 years	26(33.3)	20(76.9)	6(23.1)	Reference group	
Gender	Male	27(34.6)	25(92.6)	2(7.4)	0.32 (0.06 to 1.62)	0.171
	Female	51(65.4)	41(80.4)	10(19.6)	Reference group	
Smoking	No	62(79.5)	53(85.5)	9(14.5)	0.73 (0.17 to 3.10)	0.676
	Yes	16(20.5)	13(81.3)	3(18.8)	Reference group	
Hypertension	No	34(43.6)	27(79.4)	7(20.6)	2.02 (0.58 to 7.04)	0.269
	Yes	44(56.4)	39(88.6)	5(11.4)	Reference group	
Diabetes	No	37(47.4)	30(81.1)	7(18.9)	1.68 (0.48 to 5.83)	0.414
	Yes	41(52.6)	36(87.8)	5(12.2)	Reference group	
Trastuzumab use	No	69(88.5)	57(82.6)	12(17.4)	-	0.340
	Yes	9(11.5)	9(100.0)	0(0.0)	Reference group	
Heart disease	No	72(92.3)	63(87.5)	9(12.5)	0.14 (0.02 to 0.81)	0.029
	Yes	6(7.7)	3(50.0)	3(50.0)	Reference group	
History of percutaneous intervention	No	77(98.7)	66(85.7)	11(14.3)	-	0.154
	Yes	1(1.3)	0(0.0)	1(100.0)	Reference group	
Chronic renal failure	No	76(97.4)	64(84.2)	12(15.8)	-	1.000
	Yes	2(2.6)	2(100.0)	0(0.0)	Reference group	
Dyslipidemia	No	65(83.3)	59(90.8)	6(9.2)	0.12 (0.03 to 0.47)	0.002
	Yes	13(16.7)	7(53.8)	6(46.2)	Reference group	
Cycles number	<5	35(44.9)	30(85.7)	5(14.3)	0.91 (0.15 to 5.43)	0.924
	6=10	30(38.5)	25(83.3)	5(16.7)	1.10 (0.18 to 6.56)	0.917
	>10	13(16.7)	11(84.6)	2 (15.4)	Reference group	
Bassline ejection fraction	Normal	69 (90.8)	8 (72.7)	61 (93.8)	Reference group	0.029
	Borderline	1 (1.3)	1 (9.1)	0 (0.0)		
	Impaired	6 (7.9)	2 (18.2)	4 (6.2)		

Association between cardiac toxicity and anticancer therapy subtype

There was a higher chance of cardiac toxicity among individuals who received specific anticancer therapy subtypes, including monoclonal anticancer, alkylating agents, anthracyclines, hormonal agents, or Taxan users, as well as those who received multiple anticancer therapy subtypes (OR: 2; 95% CI: 0.57 to 6.92 and OR: 1.17; 95% CI: 0.58 to 2.35, respectively). However, these associations were not found to be statistically significant, as indicated by the p-values of 0.274 and 0.653 (Table 4).

**Table 4:** The association between cardiac toxicity and anticancer therapy subtype

Variable	Subvariable	Total (n=78) N (%)	Outcome cardiac toxicity		Univariate analysis	P-value
			No n (%) 66(84.6)	Yes n (%) 12(15.4)	OR (95 % CI)	
Monoclonal anticancer users	No	66(84.6)	57(86.4)	9(13.6)	0.47 (0.10 to 2.08)	0.324
	Yes	12(15.4)	9(75.0)	3(25.0)	Reference group	
Alkylating agent users	No	48(61.5)	41(85.4)	7(14.6)	0.85 (0.24 to 2.98)	0.804
	Yes	30(38.5)	25(83.3)	5(16.7)	Reference group	
Anthracyclines users	No	62(79.5)	53(85.5)	9(14.5)	0.73 (0.17 to 3.10)	0.676
	Yes	16(20.5)	13(81.3)	3(18.8)	Reference group	
Other agents' users	No	76(97.4)	64(84.2)	12(15.8)	-	1.000
	Yes	2(2.6)	2(100.0)	0(0.0)	-	
Taxane users	No	67(85.5)	56(83.6)	11(16.4)	0.50 (0.06 to 4.39)	0.539
	Yes	11(14.1)	10(90.9)	1(9.1)	Reference group	
Anticancer therapy subtype	No	28(35.9)	22(78.6)	6(21.4)	2.00 (0.57 to 6.92)	0.274
	Yes	50(64.1)	44(88.0)	6(12.0)	Reference group	
Number of anticancer therapies received	0	28(35.9)	-	-	1.17 (0.58 to 2.35)	0.653
	1	35(44.9)				
	2	10(12.8)				
	3	5(6.4)				

Administration of cardiac protection during treatments

Cardiac protective agents were used in 16 (20.5%) of patients and ACE inhibitors and statins were the most commonly used. In univariate analysis, statin (OR: 0.22; 95% CI: 0.05 to 0.84, P=0.028) and antiplatelet (OR: 0.19; 95% CI: 0.03 to 1.01, P=0.051) were protective agents against cardiac toxicity and were statistically significant (Table 5).

**Table 5:** The effect of cardiac protective agents on cardiac toxicity

Variable	Subvariable	Total (n=78) N (%)	Outcome cardiac toxicity		Univariate analysis	P-value
			No n (%) 66(84.6)	Yes n (%) 12(15.4)	OR (95 % CI)	
Use of cardio-protective drugs	No	62(79.5)	54(87.1)	8(12.9)	0.44 (0.11 to 1.72)	0.240
	Yes	16(20.5)	12(75.0)	4(25.0)	Reference group	
Beta-blocker	No	68(87.2)	59(86.8)	9(13.2)	0.35 (0.07 to 1.63)	0.184
	Yes	10(12.8)	7(70.0)	3(30.0)	Reference group	
ACE inhibitors	No	63(80.8)	55(87.3)	8(12.7)	0.40 (0.10 to 1.56)	0.188
	Yes	15(19.2)	11(73.3)	4(26.7)	Reference group	
Statins	No	64(82.1)	57(89.1)	7(10.9)	0.22 (0.05 to 0.84)	0.028
	Yes	14(17.9)	9(64.3)	5(35.7)	Reference group	
Antiplatelets	No	71(91.0)	62(87.3)	9(12.7)	0.19 (0.03 to 1.01)	0.051
	Yes	7(9.0)	4(57.1)	3(42.9)	Reference group	

## Discussion

Our analysis revealed that a previous history of heart disease, dyslipidemia, low baseline EF, and receiving multiple anticancer therapies were associated with cardiotoxicity occurrence. Additionally, there was an acceptable incidence rate of anti-cancer-related cardiotoxicity with precise adherence to the monitoring guidelines for cardiotoxicity. Additionally, statin and antiplatelet were protective agents against cardiac toxicity and were statistically significant. Overall, based on the utilized criteria, chemotherapy-induced cardiotoxicity was observed in 15.4% of the patients included in our study.

It is noteworthy that there is considerable variability in the incidence of cardiotoxicity among cancer patients, ranging from as low as 3.8% to as high as 37.5% in previous studies [11,12]. This variability suggests the presence of potential confounding factors or effect modifiers that contribute to the risk of cardiotoxicity. For instance, certain chemotherapeutic agents, such as doxorubicin, have been associated with higher incidences of cardiotoxicity [13]. Additionally, gender and age have also been identified as potential factors influencing the risk. Furthermore, cumulative dose, as well as electrolyte imbalances, such as low calcium or magnesium levels, have been implicated as potential contributing factors [14], warranting further investigation and control of such cases to establish correlations. Moreover, the type of malignancy appears to have an impact on the incidence of cardiotoxicity. In our study, breast and gastrointestinal malignancies exhibited the highest rates, both at 27.6%, which aligns with findings from prior studies [15,16].

Cancer and cardiovascular disease share common risk factors, including aging, physical inactivity, smoking, hypertension, diabetes mellitus, and inflammation, all of which contribute significantly to the progression and development of both entities [2,6,17]. Additionally, the presence of pre-existing cardiovascular conditions renders patients more susceptible to cardiotoxicity and other cardiac events, potentially leading to treatment modifications, dose adjustments, or even premature discontinuation of therapy [15,16,18]. In our study, a history of heart disease, impaired baseline ejection fraction, and dyslipidemia were associated with cardiac toxicity. However, no significant associations were found with age, smoking, hypertension, chronic renal failure, or diabetes. Consistent with our findings, previous studies have also reported associations between cardiac toxicity and a history of heart disease, dyslipidemia, and impaired baseline ejection fraction [16,19]. Another study reported a higher prevalence of cardiovascular events among individuals with a history of hypertension, dyslipidemia, and smoking [20]. Although our analysis did not find a significant association between smoking and cardiotoxicity, it is worth noting that smoking has been linked to increased chemotherapy toxicity and poorer overall outcomes [21]. The limited sample size of our study patients and the unknown patterns within smaller smoking groups should not be interpreted as a lack of potential association.

Similarly, our analysis did not find significant associations between age, hypertension, chronic renal failure, and diabetes with cardiotoxicity. It is important to note that various studies have reported different findings regarding age as a risk factor for cardiotoxicity, with some suggesting an increased risk in both younger and older patients, particularly in the context of anthracycline-based chemotherapy [22,23]. Furthermore, hypertension and diabetes are well-established risk factors for cardiovascular disease and have been observed to be associated with a higher incidence of cardiotoxicity among breast cancer patients receiving trastuzumab [24]. Although our study did not find a statistically significant association, it is important to consider that our sample size was relatively small, and we had a predominantly younger population, with the majority of patients being under 60 years of age.

The advancement of more potent anticancer therapy drugs has undoubtedly improved patient outcomes in cancer treatment. However, it is important to acknowledge that these drugs can also come with substantial short-term and long-term toxicities [25]. Therefore, patients who have been exposed to chemotherapeutic agents known to increase the risk of heart failure, such as anthracyclines, trastuzumab, sunitinib, and sorafenib, should undergo screening to assess the stage of their heart failure. This screening should be based on the guidelines set forth by the American College of Cardiology/American Heart Association [26,27]. In our study, we observed a higher likelihood of cardiac toxicity among individuals who received specific chemotherapy subtypes, including monoclonal anticancer agents, alkylating agents, anthracyclines, hormonal agents, or taxanes. Additionally, those who received multiple anticancer therapy subtypes also showed an increased risk, although these findings did not reach statistical significance (p=0.274 and 0.653). It is worth noting that these agents have been associated with different clinical manifestations of cardiotoxicity [28].

Ongoing research is exploring the potential benefits of administering beta-blockers, ACE inhibitors, or angiotensin receptor blockers (ARBs), as preventive measures to avoid cardiotoxicity in patients undergoing cancer treatment [16,29]. In our study, approximately 21.1% of patients received cardiac protective agents, with ACE inhibitors and statins being the most commonly prescribed. Additionally, statin and antiplatelet were protective agents against cardiac toxicity and were statistically significant. Our goal is to transition from a reactive approach to a more proactive model for preventing cardiovascular toxicities associated with cancer therapies. In line with risk mitigation strategies employed in the general population, the field of cardio-oncology can also adopt preventive measures. The concept of cardio-oncology rehabilitation represents a paradigm shift in initiating proactive efforts, particularly for cancer patients at high risk of developing cardiac dysfunction [16,26].

Study limitations

This retrospective study was conducted at a single center, which limits the generalizability of the findings to other settings. Additionally, the sample size was relatively small. Therefore, the study's results represent a subset of cancer survivors and their cardiovascular studies. Additionally, it is important to acknowledge that the criteria for defining cardiotoxicity may vary across different healthcare facilities, highlighting the lack of a universally standardized assessment. This variation makes it challenging to compare and interpret results across studies accurately. Furthermore, the study did not adjust or classify patients based on additional factors such as total cumulative dose, infusion rate, speed, or the control of cardiovascular risk factors. These factors can potentially influence the development of cardiotoxicity and should be considered in future research. To advance knowledge in this area, future large-scale studies are required to address more specific questions and establish best practices for screening cancer survivors. By considering a broader range of factors and conducting comprehensive investigations, we can enhance our understanding and improve the management of cardiovascular health in this particular patient population.

## Conclusions

Cardiotoxicity associated with anti-cancer therapy is a well-recognized phenomenon, underscoring the imperative for meticulous adherence to monitoring guidelines. Within the scope of this study, several factors have been identified as significant contributors to cardiotoxicity, including a history of prior heart disease, dyslipidemia, a low baseline ejection fraction, and the administration of multiple anticancer therapy treatments. These findings highlight the importance of proactive management strategies aimed at mitigating the potential cardiotoxic effects of anti-cancer therapies.

## References

[1] Chlebowski RT (1979). Adriamycin (doxorubicin) cardiotoxicity: a review. West J Med.

[2] Alkofide H, Alnaim L, Alorf N, Alessa W, Bawazeer G (2021). Cardiotoxicity and cardiac monitoring among anthracycline-treated cancer patients: a retrospective cohort study. Cancer Manag Res.

[3] Adhikari A, Asdaq SM, Al Hawaj MA (2021). Anticancer drug-induced cardiotoxicity: insights and pharmacogenetics. Pharmaceuticals (Basel).

[4] Perez IE, Taveras Alam S, Hernandez GA, Sancassani R (2019). Cancer therapy-related cardiac dysfunction: an overview for the clinician. Clin Med Insights Cardiol.

[5] Dong J, Chen H (2018). Cardiotoxicity of anticancer therapeutics. Front Cardiovasc Med.

[6] Johnson CB, Davis MK, Law A, Sulpher J (2016). Shared risk factors for cardiovascular disease and cancer: implications for preventive health and clinical care in oncology patients. Can J Cardiol.

[7] Vincent L, Leedy D, Masri SC, Cheng RK (2019). Cardiovascular disease and cancer: is there increasing overlap?. Curr Oncol Rep.

[8] Zamorano JL, Lancellotti P, Rodriguez Muñoz D (2016). 2016 ESC Position Paper on cancer treatments and cardiovascular toxicity developed under the auspices of the ESC Committee for Practice Guidelines: The Task Force for cancer treatments and cardiovascular toxicity of the European Society of Cardiology (ESC). Eur Heart J.

[9] Bergler-Klein J, Rainer PP, Wallner M (2022). Cardio-oncology in Austria: cardiotoxicity and surveillance of anti-cancer therapies: position paper of the Heart Failure Working Group of the Austrian Society of Cardiology. Wien Klin Wochenschr.

[10] Guglin M, Hartlage G, Reynolds C, Chen R, Patel V (2009). Trastuzumab-induced cardiomyopathy: not as benign as it looks? A retrospective study. J Card Fail.

[11] Curigliano G, Cardinale D, Dent S, Criscitiello C, Aseyev O, Lenihan D, Cipolla CM (2016). Cardiotoxicity of anticancer treatments: epidemiology, detection, and management. CA Cancer J Clin.

[12] López-Sendón J, Álvarez-Ortega C, Zamora Auñon P (2020). Classification, prevalence, and outcomes of anticancer therapy-induced cardiotoxicity: the CARDIOTOX registry. Eur Heart J.

[13] Moudgil R, Yeh ET (2016). Mechanisms of cardiotoxicity of cancer chemotherapeutic agents: cardiomyopathy and beyond. Can J Cardiol.

[14] Précoma DB, Oliveira GM, Simão AF (2019). Updated cardiovascular prevention guideline of the Brazilian Society of Cardiology - 2019. Arq Bras Cardiol.

[15] Al-Kindi SG, Oliveira GH (2016). Prevalence of preexisting cardiovascular disease in patients with different types of cancer: the unmet need for onco-cardiology. Mayo Clin Proc.

[16] Fiuza M, Magalhães A, Nobre Menezes M (2022). Clinical experience of a cardio-oncology consultation at a tertiary university hospital in Portugal: an observational study. Rev Port Cardiol.

[17] Cardous-Ubbink MC, Geenen MM, Schade KJ, Heinen RC, Caron HN, Kremer LC, Van Leeuwen FE (2010). Hypertension in long-term survivors of childhood cancer: a nested case-control study. Eur J Cancer.

[18] Bansal N, Adams MJ, Ganatra S (2019). Strategies to prevent anthracycline-induced cardiotoxicity in cancer survivors. Cardiooncology.

[19] Chavez-MacGregor M, Zhang N, Buchholz TA (2013). Trastuzumab-related cardiotoxicity among older patients with breast cancer. J Clin Oncol.

[20] Costa IB, Bittar CS, Fonseca SM (2020). Brazilian cardio-oncology: the 10-year experience of the Instituto do Cancer do Estado de Sao Paulo. BMC Cardiovasc Disord.

[21] Jassem J (2019). Tobacco smoking after diagnosis of cancer: clinical aspects. Transl Lung Cancer Res.

[22] Krischer JP, Epstein S, Cuthbertson DD, Goorin AM, Epstein ML, Lipshultz SE (1997). Clinical cardiotoxicity following anthracycline treatment for childhood cancer: the Pediatric Oncology Group experience. J Clin Oncol.

[23] Von Hoff DD, Layard MW, Basa P, Davis HL Jr, Von Hoff AL, Rozencweig M, Muggia FM (1979). Risk factors for doxorubicin-induced congestive heart failure. Ann Intern Med.

[24] Jawa Z, Perez RM, Garlie L (2016). Risk factors of trastuzumab-induced cardiotoxicity in breast cancer. A meta-analysis. Medicine (Baltimore).

[25] Mudd TW Jr, Khalid M, Guddati AK (2021). Cardiotoxicity of chemotherapy and targeted agents. Am J Cancer Res.

[26] Venturini E, Iannuzzo G, D'Andrea A (2020). Oncology and cardiac rehabilitation: an underrated relationship. J Clin Med.

[27] Piper SE, McDonagh TA (2015). Chemotherapy-related cardiomyopathy. Eur Cardiol.

[28] Rosa GM, Gigli L, Tagliasacchi MI (2016). Update on cardiotoxicity of anti-cancer treatments. Eur J Clin Invest.

[29] Kalam K, Marwick TH (2013). Role of cardioprotective therapy for prevention of cardiotoxicity with chemotherapy: a systematic review and meta-analysis. Eur J Cancer.

